# The perception of childbearing sense of coherence among Chinese couples: a qualitative study

**DOI:** 10.1186/s12889-023-17363-3

**Published:** 2023-12-02

**Authors:** Bingbing Li, Mengmei Yuan, Ke Zhang, Sha Ni, Huimin Zhao, Xi Lang, Zhenjing Hu, Tieying Zeng

**Affiliations:** 1 Department of Nursing, Tongji Hospital, Tongji Medical College, Huazhong University of Science and Technology, 1095 Jiefang Avenue, Wuhan, 430030 China; 2 School of Nursing, Tongji Medical College, Huazhong University of Science and Technology, 13 Hangkong Road, Wuhan, 430030 China; 3 School of Nursing, Shanxi University of Chinese Medicine, 121 University Street, High School Campus, Jinzhong, 030619 China

**Keywords:** Salutogenesis, Childbearing, Couples, Qualitative research, Chinese

## Abstract

**Background:**

Although childbearing health care is wellness-based and promotes normal physiology, it is in a medical model and focuses on risk aversion and disease prevention. The salutogenic theory might provide an alternative perspective to health care concerning childbearing, supporting health-promoting factors, not solely on avoiding adverse events. However, there is a dearth of qualitative research exploring couples’ perceptions of childbearing from the salutogenic lens. This study aimed to explore perceptions and experiences concerning childbearing among couples in the perinatal period and identify salutogenic aspects of it.

**Methods:**

The qualitative descriptive study adopted a directed content analysis to analyse data from a semi-structured and individual interview with 25 purposively selected Chinese couples between July 2022 and December 2022. The concepts of the sense of coherence (SOC) from the salutogenic theory were used as the theory framework for coding.

**Results:**

Definitions and content for the salutogenic aspects of Chinese couples’ perception of childbearing sense of coherence were developed. For comprehensibility of childbearing, four subthemes were extracted (the challenge to health and endurance; transition to and identification with the new role; conflict and reconciliation in relationships; resistance and compromise between social culture and personal development). For manageability of childbearing, two subthemes were extracted (helplessness and hope of childbearing; self-doubt and self-assurance of childbearing). For meaningfulness of childbearing, three subthemes were extracted (personal realisation; family bonding and harmony; the continuation of life).

**Conclusions:**

The findings of this study could give a greater understanding in maintaining couples’ health in the perinatal period from the salutogenic lens and provide a guide to further research that the salutogenic theory could bring a health and wellness-focused agenda in practice and policy-making in the perinatal period.

## Background

Couples might face deep changes in individuals’ health, roles, lifestyles, and relationships during the perinatal period. They might feel exhausted, overwhelmed, and stressed when made the demands of balance between daily life and paid work, housework, and child-rearing [1, 2]. Although some couples could feel fulfilment and satisfaction, some may encounter increased psychosocial stress, and become depressed and anxious [3]. The negative emotions of couples in the perinatal period not only have detrimental effects on themselves but also influence the psychological development of infants [4]. The parental transition brings both normative stress exposure and individual differences in the stress experience [5]. Previous studies have found that women’s understanding of their challenges, predictability and preparation of their situation, and ability to anticipate events concerning pregnancy and childbirth could help them manage pain and alleviate fear and distress during the perinatal period [6–8]. In addition, the ability to cope with adverse pregnancy events by mobilising appropriate resources could contribute to well mental health [9–11]. Moreover, a sense of meaning and purpose of childbearing may provide the motivational basis for the couples’ commitment and devotion to the parental transition [12–14]. Therefore, clarifying what changes and challenges the couples might face during the period of childbearing and improving their coping abilities and meaningfulness is vital to promote their holistic health.

The current medical system for perinatal period focuses on risk factors of disease based on pathogenesis, which uses risk prevention and avoidance to improve health [15]. A pathology-based view of fertility leads to a loss of confidence in the natural, undisturbed process of childbirth, and there is a strong link between unnecessary clinical interventions and increased morbidity and iatrogenic harm to women [16]. In this process, the term “medicalisation” has increasingly been used pejoratively to refer to the overuse of routine technical and pharmacological interventions without scientific evidence of their benefits [17]. In addition, the majority of evidence that supports the promotion of health and wellness of childbearing is framed as avoidance of risk factors and reduction of adverse outcomes, whereas little is known concerning factors that could contribute to optimum health outcomes and well-being [6, 18]. For example, the current outcome measures used for evaluating maternity care are all based on the pathogenic model and evaluate the states of illness, regardless individuals’ health conditions [19].

Salutogenesis provides an alternative to the current pathogenic model in terms of health attitudes towards perinatal care [7]. The salutogenic theory, developed by the medical sociologist Aaron Antonovsky in the late 1970s, focuses on the factors that make people healthier and happier, regardless of the objective existence of illness or disease [20]. He believes that health and well-being are determined by a person’s subjective experience of coping and dealing with stressors. According to the salutogenic theory, the individual’s health moves on a continuum from completely healthy to completely unhealthy. Stress can promote health when stressors are actively managed through the use of resources; when one’s resources and the ability to use them are insufficient to meet demand, the tension becomes stress that moves one toward the unhealthy end of the continuum [21]. The ability of people to use resources to promote health is called the sense of coherence (SOC) and it explains the extent to which people perceive their lives and stressors as comprehensible, manageable, and meaningful. The SOC has three dimensions: comprehensibility refers to one’s ability to understand the situation and to think that life is ordered and structured; manageability relates to a person’s belief that the resources needed to address challenges are available to them; meaningfulness reflects a person’s motivation to cope with stressors and see challenges as worth overcoming [20]. According to this theory, we considered that the SOC’s three dimensions could provide a theoretical lens to explore perceptions and experiences concerning childbearing among couples. However, there is a dearth of qualitative research exploring couples’ perceptions of childbearing from the salutogenic lens. In this study, we aimed to explore perceptions and experiences concerning childbearing among couples in the perinatal period and identify salutogenic aspects of it. Furthermore, a qualitative interpretation instructed by the salutogenic framework might illustrate more detailed information.

## Method

### Design

A qualitative descriptive design was used to explore perceptions and experiences concerning childbearing among couples from the salutogenic lens, as it could provide a rich description about participants’ experiences and perceptions on a phenomenon [22]. This qualitative study followed the Consolidates Criteria for Reporting Qualitative Research (COREQ) [23].

### Setting and sample

Using the purposive sampling method, potential eligible Chinese couples were recruited during their antenatal obstetric visit or waiting for delivery in the obstetric ward between July 2022 and December 2022. Participants entered the study according to education status, family income, parity, gestation status, and fertilisation method. These criteria ensured the variations in the sample to provide a broad range of information explored. The period from pregnancy to 1 year postpartum is a more dramatic transition for expectant parents/parents during the perinatal period [3, 22, 24]. Thus, the definition of childbearing in our study refers to the process from pregnancy and childbirth to child-rearing. The inclusion criteria were as follows: legal Chinese couples, during the second or third trimester of pregnancy, or within a one-year postpartum period, and being able to communicate in Chinese. Couples with psychological diseases (such as the diagnosis of depression, bipolar disorder, etc.) were excluded. Finally, a total of 25 couples were recruited for the study.

### Ethical considerations

The study protocol was approved by the Ethics Committee of Tongji Hospital (reference number: TJ-IRB20220705). Informed consent from all couples was obtained, after the explanation of the purpose and procedure of the study as well as the potential risks and rights for them to pull out of the study at any time without any influence on their medical services. Only the study team members in charge of data analysis had access to raw data, ensuring the privacy and confidentiality of participants. All interviews were conducted in a locked private room to maintain privacy and freedom from interference.

### Data collection

Face-to-face, in-depth, semi-structured interviews were used to collect the data. The first author (a PhD candidate in nursing) and an assistant researcher (a candidate for a Master of Science in Nursing) conducted the interviews. Both of them have been trained and had experience in conducting interviews. For prenatal couples, before the interview, the first author and the assistant researcher assisted the participants by providing childbearing-related information and knowledge during their obstetric visit at the antenatal clinics and obstetric wards, which could help form a relationship with the participants. In communicating with couples, the potential participants were approached according to the inclusion and exclusion criteria of this study to ensure maximum variation in the information collected. For postpartum couples, the investigators, with the help of an obstetric ward nurse, used the hospital’s obstetric electronic medical record system to locate and screen women who delivered in the hospital and met the inclusion and exclusion criteria and contacted them by telephone. The researchers introduced the content and purpose of the study to the couples and invited them to participate. The interview was conducted with the wife and husband separately at a mutually agreeable time and place (in a private room of antenatal clinics, obstetric wards, and the couple’s home). Alternatively, the participants could also choose the interview via video call. Four participants were interviewed using video calls and provided verbal consent before the interview. During the video interview, only voice was recorded. The demographics of participants were collected at the beginning of the interview.

The key questions were designed as interview guides based on the SOC from the salutogenic theory. The interview began with opening questions, such as “How do you make sense of childbearing?” “What challenges/difficulties have you experienced during childbearing?” The probing questions responding to the participant’s ideas on specific issues explored the couple’s perceptions concerning childbearing from the viewpoint of the salutogenic model, such as “What do you think about the challenge/difficulty?” “What are your feelings/perspectives about it?” “What resources are being used to deal with it?” “What are your feelings/perspectives at that time?” “In what aspects are childbearing important?”

All interviews were tape-recorded, each lasting 45–90 minutes. They were conducted in Chinese, and the content quoted in this article was translated into English. Field note memos were taken to record the non-verbal expressions. When data saturation was attained with 23 couples, two additional couples were interviewed and no new codes appeared. We interviewed each individual once.

### Data analysis

Because the analysis sought to explore perceptions and experiences concerning childbearing among couples from the salutogenic lens, a directed content analysis approach was used for coding the data in this qualitative study. Directed content analysis draws from theories or existing coded categories, but does not exclude the possibility of generating new categories or themes during the analysis process, which is also useful to focus the research question and test a pre-existing theory in a different situation [25, 26]. The SOC from the salutogenic theory was used to guide the directed content analysis process: comprehensibility, manageability, and meaningfulness. If additional themes arose, they were allowed to emerge from the analysis process. All interviews were transcribed verbatim. Simultaneous collection and analysis of data mutually shaped each other. Nvivo 12.0 was used to encode the text of all the interviews. The first author and corresponding author read the transcribed data as well as coded and created the categories independently. Then, the findings were compared and discussed by the two authors until a consensus was reached on themes and subthemes.

### Trustworthiness

In order to improve credibility, a pilot testing of the interview was conducted on two potential couples to improve the interviewer’s communication skills and the interview guide. Two researchers (the first author and the corresponding author) analysed the data independently. If there is a dispute during the process, another expert would be invited to participate in the discussion until a consensus is reached. The extracted codes and subcategories were shared with 15 couples. In order to ensure dependability, the study gave an in-depth description of the design. Although guided by the salutogenic theory, researchers were flexible and open to processes and themes. We collected data until no new information and codes emerged to guarantee the saturation. To ensure confirmability, researchers discussed with peers and experts weekly from the beginning of the design to the end of the data analysis. An audit trail was created to document steps (such as interview data, data-collecting, coding and decision-making memos). To facilitate transferability, we described the context, sampling strategy, selection and characteristics of participants in detail. Comprehensive descriptions and appropriate quotations were used to enrich the findings. We discussed how the findings resonated with the existing literature.

## Results

A total of 25 couples participated in the study. The mean age of the wives and their husbands were 31.6 years (standard deviation (SD) = 2.7) and 33.0 years (SD = 4.6), respectively. Most couples have completed college or higher education. 80.0% of the wives and 100% of the husbands were employed. Details of the demographic and obstetric characteristics are presented in Table 1.

**Table 1:** characteristics of participants (*N* = 50)

Characteristics	Mothers (*n* = 25) *n*(%)	Fathers (*n* = 25) *n*(%)
Age, mean (SD)	31.6 (2.7)	33.0 (4.6)
Education		
Junior high school or lower	3 (12.0)	3 (12.0)
Senior high school	7 (28.0)	3 (12.0)
College or higher	15 (60.0)	19 (76.0)
Employment status		
Unemployed	5 (20.0)	0 (0)
Employed	20 (80.0)	25 (100)
Monthly household income (¥)		
≤ 3000	7 (28.0)	1 (4.0)
3001–5000	4 (16.0)	1 (4.0)
5001–10,000	9 (36.0)	14 (56.0)
> 10,000	5 (20.0)	9 (36.0)
Parity		
Nulliparous	9 (36.0)	
Multiparous	16 (64.0)	
Gestation status		
Second trimester	6 (24.0)	
Third trimester	8 (32.0)	
Postpartum (within 1 month)	4 (16.0)	
Postpartum(1–6 month)	4 (16.0)	
Postpartum(6–12 month)	3 (12.0)	
Fertilization method		
Spontaneous conception	19 (76.0)	
Assisted reproductive technology	6 (24.0)	

**Fig. 1:**
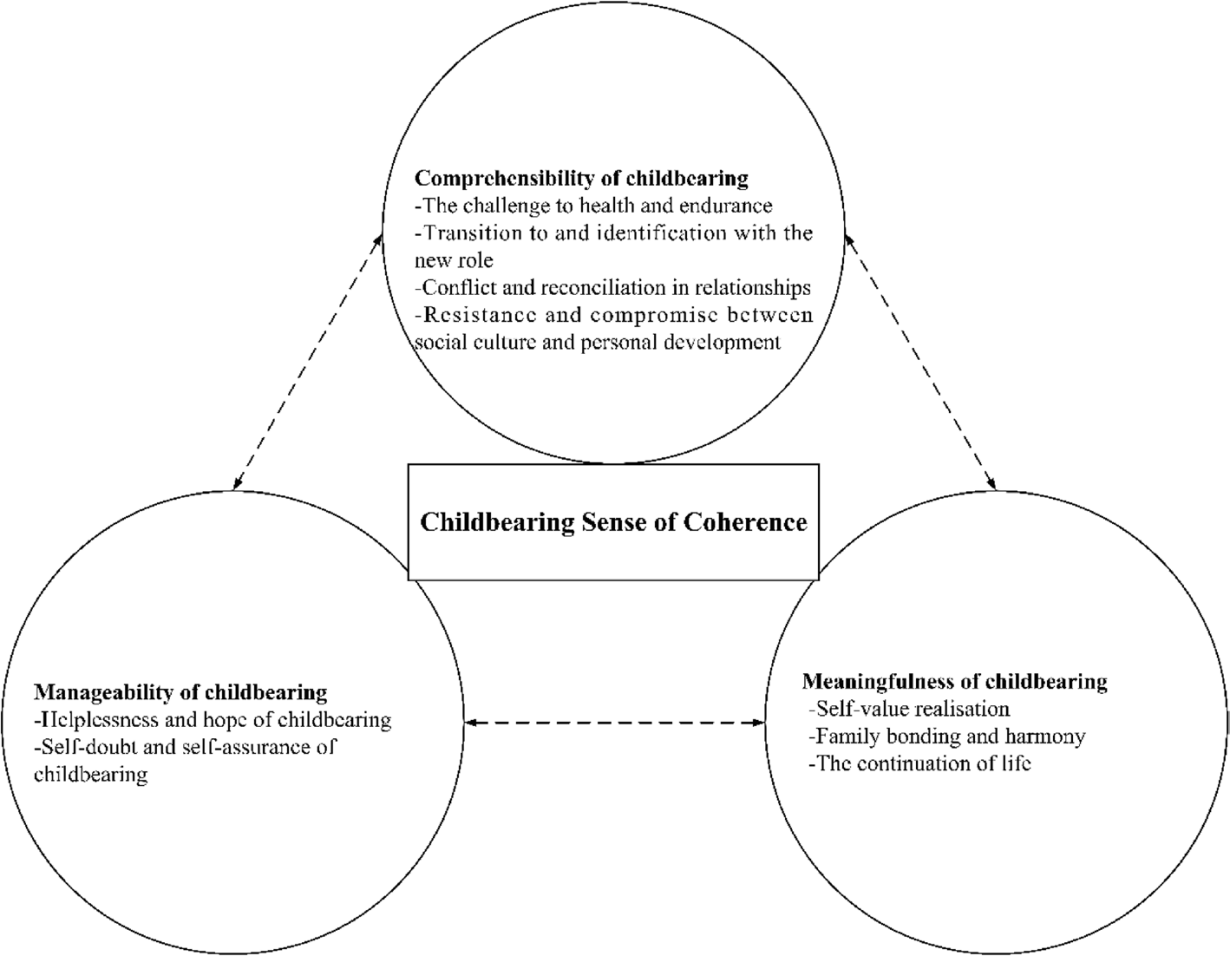
A salutogenic model of the childbearing sense of coherence

Three major themes and nine subthemes emerged from the qualitative study regarding the couples’ perceptions of the childbearing sense of coherence. These themes and subthemes are illustrated in Fig. 1.

### Comprehensibility of childbearing

Comprehensibility of childbearing refers to the extent to which the individual regards the stimuli from the internal and external environments during childbearing as reasonable and acceptable. Most couples expressed that their health status, roles, relationships, and personal development have changed. Their personal lives were disrupted by these changes in different extents. Four subthemes were identified: the challenge to health and endurance, transition to and identification with the new role, conflict and reconciliation in relationships, and contradiction/resistance and compromise between social culture and personal development.

#### The challenge to health and endurance

The couples expressed that they have undergone many changes during childbearing, including changes in the wife’s physical appearance, pregnancy reactions, and declined sleep quality. In addition, they faced the unknown of delivery and risks to the infant. Some couples accepted the changes in an emotional swing, while others expressed worries and fears.

It is painful every day. I just go through it day after day, and then one day I just stop throwing up. Then I never threw up again (smile). This kind of physical discomfort might be common among pregnant women because it is a stage of life. (P4, wife, first-time parent, third trimester of pregnancy)

I feel like my body has changed so much, which affects me a lot. Especially after birth, I don't think my body can recover. After giving birth, I felt so stressed that I wanted to lose weight. What I feel most annoying is that my husband always thinks my body has changed, as if he is consciously reminding me to lose weight. I have children for him, to a large extent, and I feel like I've done something for my family. Nevertheless, he can't understand me, which makes me think that maybe society is just too demanding of women in this respect. You have to have a baby as well as care about your image. (P19, wife, second-time parent, postpartum 10th month)

My wife and I didn't sleep much for the first two months after the baby was born. When we could sleep, we fed the baby intermittently every two hours. The first year, I was impressed with sleep and rest. If you don't sleep well, you will be in a bad state of mind, especially during the daytime. I think my memory has lost a lot in recent years, and I guess it is associated with my sleep…I have lost a lot of hair, my health is not as good as it used to be, and my stomach and intestines have become worse. (P14, husband, second-time parent, second trimester of pregnancy)

#### Transition to and identification with the new role

The transition of the role means the assumption of responsibility. Some couples were stressed by the feeding, education, relationship balance, as well as the financial burden that comes with them during childbearing.

I want to educate the kids well. When she/he encounters something, you have to teach him what’s right and wrong, as well as outlook on life and values. How are you going to teach her/him to be polite, honest, kind and to have a sense of social responsibility? Sometimes, my ideas may not be right. When the child encountered this issue, how do you teach your children…it makes me feel pressured. (P21, wife, first-time parent, postpartum 8th month)

I'd be more stressed, and there's a huge difference between having a kid and not having one. It is said that the children raised are beasts of gold. After all, the kid is my flesh and blood. I certainly want to give the best to him/her. The most direct affirmation is to raise the income…The most obvious change is that I only consider taking care of our parents and wife in the past, but now there are too many unstable factors with a kid. (P14, husband, second-time parent, second trimester of pregnancy)

I have an older son, so I feel a little guilty and ashamed because I had less care for him since I became pregnant. Balancing two kids was hard for me, and it’s still the hardest thing. For example, I have to take care of my younger child, but I have no time to tutor my older son on homework. In addition, there is no time to take care of his mood. He is at the age to constantly share with you some things, such as things that happened in school and his thoughts, but I don't have too much time to deal with these. (P19, wife, second-time parent, postpartum 10th month)

Some couples regarded the parental transition in roles and responsibilities as a new turning point and an opportunity to grow. They expressed that they could rethink and position their roles and responsibilities.

I feel like I've grown up a lot since having kids. Without my children, I always felt like I was a child. I think I had a strong sense of responsibility after the child was born. Because our parents are not around to assist us, we rely entirely on ourselves. We were from a bewildered state to know how to deal with it, and there was a significant growth since then. (P14, husband, second-time parent, second trimester of pregnancy)

### Conflict and reconciliation in relationships

The arrival of a new baby is usually a joyful event for the whole family, but the relationship between family members will change. Some couples will quarrel with their parents or spouse due to the inconsistent conception and method concerning childbearing, which could make them fall into the struggle of negative emotions.

I feel like it's my job to have a baby, and I'm the tool of them to have a baby. Getting pregnant and giving birth seems to be my responsibility and fault from the beginning. They all say how careless I am. I feel that the whole world loves my child very much, just as I do not love my child. (P3, wife, first-time parent, third trimester of pregnancy)

My mom is babysitting for us. I don't agree with some of her views on the education of children. Old people dote on the children. On the issue of taking care of children... My mother was around us, and actually it was very upsetting for me during the kids growing up, because the old people were too doting. (P19, husband, second-time parent, postpartum 10th month)

In the process of overcoming relationship conflicts in the family, some couples expressed that they come to understand and accept the mixed realities of family life that come with childbearing.

Many new conflicts, challenges and joys are bound to arise in the small family, as well as in the extended family. There will be a lot of happiness, and meanwhile headaches. (P4, wife, first-time parent, third trimester of pregnancy)

Life has changed a lot from what it used to be. I have thought of both the good and the bad. Conflicts will definitely increase, but so will your happiness. And then you're bound to have a lot of dilemmas that you wouldn't have had before. Children bring a lot of happiness to the family, but also trouble. Life is both happy and sad. (P23, husband, second-time parent, postpartum 5th month)

#### Resistance and compromise between social culture and personal development

Some couples expressed that childbearing took up too much time and energy, hindering the development of careers, hobbies, etc.; they embraced this transition in a pendulum of confrontation and concession.

You're working less, and you're actually anxious. Everyone else is running hard, but you are still slow...so, even though you feel better due to reduced physical intensity, you feel less happy and less competitive in the workplace. However, there is nothing to be done about this. For now, you can only take care of one aspect. (P4, wife, first-time parent, third trimester of pregnancy)

How do you divide your time? Since the baby is born, who will take care of him/her? Issues like future company, and the regulation of your work hours…children have to have physical examinations, vaccinations…how to spend more time with him/her, this will pose some challenges to your work. Life is a balancing act, on your work or your life, etc. What you choose to give up or get depends on what you want more, which also means you have to give up something. (P24, husband, first-time parent, postpartum 4th month)

Some wives said that the cultural demands on women are both being a good nurturer and an excellent worker in the workplace, which put them on the warpath between social culture and personal development.

I have thought about whether I could quit my job and take care of the kids at home, but it would be like losing my value suddenly. No one pays you, and my husband may not think I have made contributions…Especially my mother-in-law does not take care of the children carefully, resulting in the child's safety risks…I struggled to think about it. However, the answer in my heart told me that I definitely could not resign…I should not lose myself. Because society demands too much on women, if you are totally attached to men, the consequences may be very sad. (P19, wife, second-time parent, postpartum 10th month)

I have sacrificed all my time to take care of the kids. I felt like my personal development had almost stopped…I have no time to go out to play or sign up for a hobby class. I am completely trapped by the child. That's what pregnancy is like, because you can't move around and you can't do much. (P24, wife, first-time parent, postpartum 4th month)

### Manageability of childbearing

Manageability of childbearing refers to the extent to which the individual utilises internal and external resources to cope with challenges from childbearing. Two subthemes were identified: helplessness and hope of childbearing and self-doubt and self-assurance of childbearing.

#### Helplessness and hope of childbearing

Many couples expressed that they will do some preparation in psychological, knowledge, skills and materials concerning childbearing in advance and will also carry out active behavioural regulation. For instance, the wife adjusted her life status, and the spouse actively participated in the pregnancy and tried to meet the needs of the wife. Other couples said that the changes during childbearing were out of their control and they had to endure.

I have prepared for the changes in my body, as well as the pain of childbirth in mind, because I have anticipated these ahead of time. I think it is just a matter of tolerance. I don't feel a lot of pressure. (P7, wife, first-time parent, second trimester of pregnancy)

Every time I tried to come with her (my wife) for a prenatal examination. I felt that it would make her feel more secure and confident…it would be a kind of encouragement for her. (P4, husband, first-time parent, third trimester of pregnancy)

Sometimes I feel bad because I think she's pregnant and the months are older… she's under a lot of pressure at work, but I can't do anything to help. All of these make me feel bad (sigh). (P1, husband, first-time parent, second trimester of pregnancy)

Especially when I gave birth and was doing the month, my whole body was swollen. Once when I got out of the shower and saw myself in the mirror, I wanted to cry (sob). I can’t believe I was looking like this, but I still had to feed. I felt like my body didn't belong to me, you know? I am out of shape and have to raise the next generation. It's a long story. (P25, wife, first-time parent, postpartum 6th month)

Some couples said that their own direct experience and the vicarious experience of others provided an important reference. Some couples expressed that they felt overwhelmed when it came to pregnancy-related difficulties.

I was just talking to some pregnant women at the door. I'm sure I don't have as many concerns as they do. Relatively speaking, I know what it's like to be pregnant. For example, when I buy a maternity package, I will be very targeted. I know exactly what I need. I will not buy it if I don’t need it in a hurry. I think second-time mothers have a lot more experience than first-time mothers. (P13, wife, second-time parent, third trimester of pregnancy)

My wife felt the baby kicked her and made her uncomfortable. There was pressure on her inside and she was uncomfortable… but I couldn't help at that time, so I was anxious. I want to help her, but I can't. I don't know how to make her feel better. (P5, husband, first-time parent, second trimester of pregnancy)

Parents and spouses were the most frequently mentioned sources of social support. The couples said that mutual communication, assistance and companionship with spouses can help them cope with the challenges during childbearing and thus relieve stress. However, some wives reported their spouses were in passive involvement. They perceived very limited support from their families and, therefore, felt powerless in the face of the pressures of childbearing.

My parents have been very supportive. I just didn't expect anything more (Laughter). They would take care of me in my diet and daily life. I didn't expect anything more. (P7, wife, first-time parent, second trimester of pregnancy)

In the beginning, I was a little anxious, but my husband was very helpful. I usually communicated with him. I think he is very helpful, and we will not have a lot of pressure in the financial aspect. We will make plans after the birth of the child. He always says to me, “Don't worry”. (P2, wife, first-time parent, third trimester of pregnancy)

They don't think it bothers me. They don't understand. They don't think you have postpartum depression. They think you're already well taken care of, and why would you do that? (P13, wife, second-time parent, third trimester of pregnancy)

I feel that my husband is not in line with me in this regard, such as properly communicating with his mother, telling her what to do, looking for a nanny, or inviting a relative to look after the baby. He made me feel like if I had to do this, I would have to pay the fiddler. He had no intention of taking the consequences with me, and it never worked out anyway. (P19, wife, second-time parent, postpartum 10th month)

Many couples expressed that medical social supports enabled them to obtain more authoritative information resources and security. In addition, they said that friends, relatives, colleagues and leaders in the workplace can help them solve some problems during childbearing effectively. However, other couples reported not being able to get external help when dealing with childbearing-related stress.

I had regular antenatal visits and was assessed by the same doctor because he has a better understanding of my condition. I followed the doctor's advice. I don't think it's going to be anything wrong. I have regular check-ups and learn about childbearing information in time. I have no anxiety. (P2, wife, first-time parent, third trimester of pregnancy)

I posted a message on social media saying I felt sick, or I asked people what brand of milk powder, baby clothes, or diapers were better to buy. High school classmates whom I haven't contacted for years sent me a message, cared about me, and then told me what brand to buy. Some classmates who had babies said they had extra ones at home and would send them to me (smile). (P5, wife, first-time parent, second trimester of pregnancy)

The leaders of my workplace are very kind. They know my condition (difficulty conceiving). So, they haven't assigned me strenuous work since I got pregnant. If I don't feel well, I can rest at home. I have nothing to worry about. Usually, when they need me to do something, they will call me. My colleagues also help me share a lot of work. (P10, wife, first-time parent, second trimester of pregnancy)

I don't think you can get much help from outside for such problems. But this kind of family chores slowly accumulates over time. It's like you can't put your finger on what the problem is when you talk to others. (P20, wife, first-time parent, postpartum 1st year)

Some wives said that their choices about childbearing are influenced by social norms. Because they were worried that not having children would be regarded as a kind of behavior against social norms, they passively made the choice to have children to conform to social norms.

I didn't want to have children. I thought they would be noisy…I probably couldn't handle it, so I never really wanted to have kids of my own. Then I thought everyone has kids, and my husband loves kids. That's why I'm gonna have a baby. That seems to be the case for a lot of people... I can't understand or accept... We haven't had children since we've been married for so long that people might think there's something physically wrong with me or my husband. There's pressure. People keep asking. (P5, wife, first-time parent, second trimester of pregnancy)

#### Self-doubt and self-assurance of childbearing

On the one hand, the couples expressed that their own or others’ positive experience of childbearing will strengthen their childbearing self-efficacy; therefore, they will have enough confidence in their ability to cope. On the other hand, they said that they would not believe in their ability to cope with childbearing-related difficulties because of their own or others’ negative experiences.

If you don't do something in person, you will not know what you encounter. After my second child was born, we are not under a lot of pressure concerning childbearing. We are fully at ease. (P14, husband, second-time parent, second trimester of pregnancy)

I watched my friend nurturing and companying his child from a very young age, and I wanted to experience it. I would feel very happy to see my children grow up day by day…it makes me happy just imagining the picture. (P5, husband, first-time parent, second trimester of pregnancy)

I was going to choose the caesarean section. The doctor suggested a natural delivery. However, I am still a little worried about the pain…because a colleague of mine chose anaesthetic pain relief and natural delivery…the labour process was too long, and the baby suffered from aspiration pneumonia and was hospitalised in the pediatric department for a long time… another colleague went through a day of vaginal labour and ended up having a cesarean delivery. (P12, wife, second-time parent, third trimester of pregnancy)

I'm just worried that it's going to be hard to have a normal labour now with my second child, as I didn't have a very good experience with my first. (P14, wife, second-time parent, second trimester of pregnancy)

### Meaningfulness of childbearing

Meaningfulness of childbearing refers to the extent to which the individual recognises the meaningfulness of everything experienced during childbearing and is willing to put effort into it. Most couples perceived childbearing with a new sense of meaning, which is the basis of their motivation to cope with challenges brought by childbearing. This study revealed three subthemes on the meaningfulness of childbearing: personal realisation, family bonding and harmony, and the continuation of life.

#### Self-value realisation

Many couples mentioned that childbearing is a unique experience for them, and a child is a new living individual. When they accompany the kids to growth, they can experience and comprehend life from different angles. In addition, they said the birth and upbringing of children could enrich and complete life.

Your life is more complete because of your children ...... They make your life fuller, more prosperous and better from different perspectives. (P4, husband, first-time parent, third trimester of pregnancy)

I actually think raising a child means you grow up with her/him, and then she/he leaves you. It's a journey through life, and you give each other a good memory and then leave ...... (P4, wife, first-time parent, third trimester of pregnancy)

Many couples said that they take life more seriously and work harder as they devote themselves to their children. They also have a deeper understanding of the responsibility of being parents.

I'm especially grateful to my older children. Because ever since I had her, I think I've just started to grow, and there's been a lot of transformation. I used to think of myself as a child and then slowly take on the responsibilities of a mother. I think having a second child may be another opportunity for me to grow up better and experience the role of a mother deeply, including my new plan and career development. I will have some new thinking. All of these are good opportunities to help me grow better. (P13, wife, second-time parent, third trimester of pregnancy)

Some couples said that children are the hope of their lives and can satisfy their emotional needs.

People always need to have some sustenance when they are alive. When you're young, you can focus on superficial things, like playing games. Or when you entered the workforce during the first few years, you can concentrate on your work. Over time, you always need a more stable soul to rest on… it could be your relationship, and later it could be having a child. (P2, husband, first-time parent, third trimester of pregnancy)

#### Family bonding and harmony

Many couples said that children are important emotional bonds for both the extended family and the couple. While children bring laughter and happiness to the family, they can make the relationship between husband and wife and the relationship between the couple and their parents more united and intimate.

After all, children bring a lot of happiness to the family, and the old must be very happy in the extended family…they look at kids just like they could see us when we were kids. (P4, husband, first-time parent, third trimester of pregnancy)

When the eldest child was born, I became more connected to my wife. There's more than just love between us. And now we're working together to raise this baby. (P14, husband, second-time parent, second trimester of pregnancy)

#### The continuation of life

Many couples said that having children is the natural law of human childbearing and a continuation of their own lives.

After having a child, you can teach him/her some of your good ideas, which is also a continuation of your life. (P10, husband, first-time parent, second trimester of pregnancy)

This is the law of nature, because all living body is in the process of childbearing. Humans are higher mammals than other animals; childbearing is something you are born with…it's a natural choice…it's the ability that you have. (P7, husband, first-time parent, second trimester of pregnancy)

## Discussion

In this study, the childbearing sense of coherence provided a holistic conceptual model for couples in perinatal period from salutogenic aspects. It is unique in its depth in exploring the concept and content of comprehensibility, manageability and meaningfulness concerning childbearing. Comprehensibility, manageability and meaningfulness concerning childbearing reflect cognitive, behavioral, and emotional aspects of couples’ perinatal health. Comprehensibility of childbearing refers to the extent to which the individual regards the stimuli from the internal and external environments during childbearing as reasonable and acceptable. Manageability of childbearing refers to the extent to which the individual utilises internal and external resources to cope with challenges from childbearing. Meaningfulness of childbearing refers to the extent to which the individual recognises the meaningfulness of everything experienced during childbearing and is willing to put effort into it.

We found that in the comprehensibility of childbearing aspects when exposed to the internal and external stimuli, most couples expressed that their health status, roles, relationships, and personal development have changed. They felt that their personal lives were disrupted by these changes to different extents. We described them as the challenge to health and endurance, transition to and identification with the new role, conflict and reconciliation in relationships, and resistance and compromise between social culture and personal development. The findings that both wives and husbands in this study have been disrupted by the changes in health, roles, and relationships brought by childbearing are consistent with the results of a study conducted among couples during the parental transition in Hong Kong China [12]. In this study, we paid attention to perceptions of childbearing among couples, which was a remedy to previous studies which focused solely on women or men. Both men and women are affected by the challenges of childbearing. As couples interact as a cohesive unit, it is of great value to understand how each partner feels about childbearing. It is worth noting that we found that not only wives would face health challenges and changes during the perinatal period, but also husbands would express changes in health status and concerns about the health status of the baby and the wife. This reminds us that we should also pay attention to changes in male health during childbearing. Some previous studies have found that couples’ cognitive evaluation of challenges encountered during the parental transition could induce stress, irritability, and fatigue [2, 27]. Therefore, it is of great significance to clarify the acceptable and understanding degree of the couples to the challenges of childbearing to formulate corresponding intervention programs for the comprehensibility of childbearing, thus promoting their health level.

In accordance with the results of previous studies, work and family conflicts represented a pervasive experience of contemporary Chinese couples [28]. Our study showed that resistance and compromise between social culture and personal development were especially obvious in wives. The reason might be that, with the reform of the social economic system and ideology, women in China have assumed more social roles. The gender role has changed qualitatively from “men taking charge of the outside and women taking charge of the inside” to “men and women sharing responsibility”. Currently, the majority of women in China are engaged in full-time employment, which is hard for them to meet the demands imposed by their dual roles as workers and mothers [29]. Understanding the potential effects of pregnancy and childbirth in advance may help women to manage the impact of these changes and help them to cope better during this transitional period. In recent years, the Chinese government has issued documents recommending the development of childbearing-friendly workplaces and optimised employee management practices, allowing employees to balance their work and childbearing responsibilities [30]. For instance, a series of policies have been established to support gestation. The population and family planning regulations of many provinces and cities across the country also specify that spouses are entitled to paternity leave or nursing leave, supporting couples to share the responsibility of childbearing jointly.

Manageability of childbearing is the behavioral aspect and refers to the extent to which internal and external resources are utilised in coping with challenges from childbearing. In this study, the second-time parents said that their own direct experience could provide an important reference to the childbearing of this time. The childbearing experience accumulated by the second-time parents is their primary internal resource used to cope with challenges during this period, which is in consistent with findings of previous research [19]. Support from families, especially spouses and parents, was mentioned as the most important external resource in coping with challenges from childbearing. Unlike past generations, fathers nowadays are expected to be one of the family’s breadwinners as well as to be involved in sharing responsibility for gestation, care, and upbringing of the children. In line with previous findings, our study found that fathers in contemporary Chinese society were more willing to participate in childcare and share parental responsibilities with their partners [31]. Childbearing is a responsibility that couples as an intimate union need to undertake together. Therefore, support from the husband is undoubtedly the most important external resource for a wife in the perinatal period. We also found that parents or parents-in-law of couples play an important role in assisting in taking care of children in the extended family. This phenomenon is unique in China and could stem from Chinese collective culture, which emphasises family ties and manifest as three generations living under the same roof [32]. Collective coping originating from the culture of collectivism is still prevalent and practiced in Chinese society [33]. The collective welfare of the family is emphasised, so in the extended family, the parents of the couples are obligated to take care of their grandchildren and subordinate their own interests and desires. In addition to support from spouses and parents, the couples also sought information and advice concerning childbearing from their friends, colleagues, and healthcare professionals, which was in accord with other findings [12, 34]. These findings suggested that improving couples’ internal resources, such as experience, and external resources, like support from family, are critical for coping with the challenges of childbearing. Identifying and mobilising these resources requires not only the efforts of individual mothers or spouses, but also the support of family members, society and national policies.

Meaningfulness of childbearing is the emotional or spiritual aspect and refers to the recognition of the meaning of childbearing and the extent to which effort is devoted to it. Meaningfulness is considered as the motivational element of SOC, which provide people with care and the ‘importance of being involved in challenges [20]. Consistent with the salutogenesis, the perception of the meaningfulness of childbearing motivated couples to overcome difficulties. In this study, we found that couples regard childbearing as valuable regarding personal realisation, family bonding and harmony, and natural law. SOC theory suggests that the “meaningfulness” of an event will determine people’s comprehension of the event and their willingness to commit resources to it [20]. As the meaning of childbearing provides the drive and motivation for couples to be positive, efforts need to motivate couples’ realisation of the meaningfulness of childbearing so that they will experience a more ‘predictable’ and ‘manageable’ process.

### Strengths and limitations

Participants were varied in education status, family income, parity, gestation status, and fertilisation method, which could maximise the validity of the findings. In this study, the content for the perception of childbearing sense of coherence were developed in Chinese couples. Considering the influence of different cultures, the content of childbearing sense of coherence may vary to some extent in different regions. Future studies could explore the content of couples’ perception of childbearing sense of coherence in different countries and regions and compare their similarities and differences. Furthermore, the influence factors of the perception of childbearing sense of coherence could be explored.

## Conclusions

This study explored perceptions and experiences concerning childbearing among couples in the perinatal period and identified salutogenic aspects of it. This exploration may help us better understand couples’ cognitive, behavioral, and emotional and spiritual experiences of childbearing, as well as enrich the salutogenic framework in the area of childbearing. The findings of this study could give a greater understanding in maintaining couples’ health in the perinatal period from the salutogenic lens and provide a guide to further research that the salutogenic theory could bring a health and wellness-focused agenda in practice and policy-making in the perinatal period.

## Data Availability

The data used in the current study are available from the corresponding author upon reasonable request.

## Abbreviations

SOC: Sense of Coherence
COREQ: the Consolidates Criteria for Reporting Qualitative Research
